# Nanoparticle as a novel tool in hyperthermic intraperitoneal and pressurized intraperitoneal aerosol chemotheprapy to treat patients with peritoneal carcinomatosis

**DOI:** 10.18632/oncotarget.20596

**Published:** 2017-08-31

**Authors:** Maciej Nowacki, Margarita Peterson, Tomasz Kloskowski, Eleanor McCabe, Delia Cortes Guiral, Karol Polom, Katarzyna Pietkun, Barbara Zegarska, Marta Pokrywczynska, Tomasz Drewa, Franco Roviello, Edward A. Medina, Samy L. Habib, Wojciech Zegarski

**Affiliations:** ^1^ Chair of Department of Surgical Oncology, Ludwik Rydygier Collegium Medicum in Bydgoszcz, Nicolaus Copernicus University in Torun, Oncology Centre of Franciszek Łukaszczyk Memorial Hospital, Bydgoszcz, Poland; ^2^ Department of Plastic and Reconstructive Surgery, Wake Forest School of Medicine, Winston-Salem, NC, USA; ^3^ Chair of Urology, Department of Regenerative Medicine, Ludwik Rydygier's Collegium Medicum in Bydgoszcz, Nicolaus Copernicus University in Torun, Toruń, Poland; ^4^ Department of General Surgery (Peritoneal Surface Surgical Oncology), Fundación Jiménez Díaz Hospital, Madrid, Spain; ^5^ General Surgery and Surgical Oncology Department, University of Siena, Siena, Italy; ^6^ Department of Surgical Oncology, Medical University of Gdansk, Gdansk, Poland; ^7^ Chair of Cosmetology and Aesthetic Dermatology, Ludwik Rydygier Collegium Medicum in Bydgoszcz, Nicolaus Copernicus University in Torun. Bydgoszcz, Poland; ^8^ Department of Pathology, University of Texas Health, San Antonio, TX, USA; ^9^ Department of Cell Systems and Anatomy, University of Texas Health Geriatric Research Education, San Antonio, TX, USA; ^10^ South Texas Veterans Health Care System, San Antonio, TX, USA

**Keywords:** CRS + HIPEC, HIPEC, PIPAC, peritoneal carcinomatosis, nanomedicine

## Abstract

The treatment of peritoneal surface malignances has changed considerably over the last thirty years. Unfortunately, the palliative is the only current treatment for peritoneal carcinomatosis (PC). Two primary intraperitoneal chemotherapeutic methods are used. The first is combination of cytoreductive surgery (CRS) and Hyperthermic IntraPEritoneal Chemotherapy (HIPEC), which has become the gold standard for many cases of PC. The second is Pressurized IntraPeritoneal Aerosol Chemotheprapy (PIPAC), which is promising direction to minimally invasive as safedrug delivery. These methods were improved through multicenter studies and clinical trials that yield important insights and solutions. Major method development has been made through nanomedicine, specifically nanoparticles. Here, we are presenting the latest advances of nanoparticles and their application to precision diagnostics and improved treatment strategies for PC. These advances will likely develop both HIPEC and PIPAC methods that used for *in vitro* and *in vivo* studies. Several benefits of using nanoparticles will be discussed including: 1) Nanoparticles as drug delivery systems; 2) Nanoparticles and Near Infrred (NIR) Irradiation; 3) use of nanoparticles in perioperative diagnostic and individualized treatment planning; 4) use of nanoparticles as anticancer dressing’s, hydrogels and as active beeds for optimal reccurence prevention; and 5) finally the curent *in vitro* and *in vivo* studies and clinical trials of nanoparticles. The current review highlighted use of nanoparticles as novel tools in improving drug delivery to be effective for treatment patients with peritoneal carcinomatosis.

## INTRODUCTION

The treatment of peritoneal surface malignancies has evolved extraordinarily over the years from cyto-reductive surgery (CRS) techniques through intraperitoneal drug delivery to intraoperative chemotherapy with hyperthermia (HIPEC) and/or post-operative normothermic chemotherapy (EPIC) [1]. One of the most recent evolving intraperitoneal delivery systems is PIPAC (Pressurized IntraPeritoneal Aerosol Chemotheprapy) [2, 3], which is performed with palliative intent or before HIPEC in selected cases [4]. It is also used as intraperitoneal neoadjuvant therapy that can be combined with systemic neoadjuvant chemotherapy to induce responsiveness with CRS+HIPEC to treat with curative intent patients. The cytostatics do not remain in the abdominal cavity for prolonged periods of times particularly small molecular weight chemotherapeutics, which are quickly absorbed into the circulation [5, 6]. The ideal drug for intraperitoneal administration should remain active in the peritoneal cavity for prolonged period of time to avoid systemic absorption and toxicity. In addition, it should be a selective target for the tumor cells with deep penetration into tumor nodules [7, 8]. Currently, most of the HIPEC, EPIC and PIPAC procedures are using the intravenous formulation of chemotherapeutic agents.

The rationale for using nanoparticles (NPs) in the treatment of peritoneal carcinomatosis is to take advantage of the anti-cancer therapy as potential target for longer effectiveness in the peritoneal cavity. The high intracellular concentrations of nanoparticles can be achieved because they are able to enter the cells without being recognized by P-glycoprotein, which is one of the most important mechanisms in drug resistance [9]. Nanoparticles work as carriers of chemotherapeutics selectively target the tumor cells [10]. Twelve nanomedicines are clinically approved as anti-cancer drugs but their intraperitoneally use has not been reported [11]. However, the peritoneum can be targeted with NPs [12, 13] and EPIC can be performed using NPs carrying chemotherapeutics as metronomic therapy. The NPs can also be used for HIPEC or PIPAC. The depot systems such as hydrogels have some problems of homogeneus distribution and tolerance was investigated to achieve the sustained release of NPs.

The large surface area of the peritoneum, presence of adhesions, mucus, fluid and effects of gravity make intraperitoneal delivery of chemotherapy challenging. Many of the intravenous formulations are currently use the off-label for HIPEC and PIPAC which are susceptible to rapid clearance, exhibit local toxicity have limited penetration depths [14]. Nanoparticles with their sub-micron size, versatility of physical, chemical properties and easily modifiable surface are uniquely poised to bypass the body clearing systems. They penetrate through extra- and intracellular barriers to deliver drugs into cancer cells thereby enhancing chemotherapeutic efficacy. Specific manipulation of the chemical composition of nanoparticles can significantly increase peritoneal residence time, thereby prolonging exposure of tumor to chemotherapeutic agents will increase the local drug concentration is primary goal of intraperitoneal chemotherapy [15].

The latest advances in the field of nanoparticles and their application to precision diagnostics and improved treatment strategies for peritoneal carcinomatosis (PC) will be discussed. These advances will likely develop both the HIPEC and PIPAC methods that were tested in *in vitro* and *in vivo* studies. We will discuss: 1) Nanoparticles as drug delivery systems; 2) Nanoparticles and Near Infrred (NIR) Irradiation; 3) use of nanoparticles in perioperative diagnostic and individualized treatment planning; 4) use of nanoparticles as anticancer dressing’s, hydrogels and as active beeds for optimal reccurence prevention; and 5) finally the current *in vitro* and *in vivo* studies and clinical trials of nanoparticles.

### Nanoparticles as drug delivery systems

Nanocarriers are the epitome of rational drug design with advancements in material science allowing for the manufacture of nanoparticles optimized for desired mode of delivery, location within the body, and characteristics of the tumor. These advantages offer an exciting prospect for improving the therapeutic index of chemotherapy drugs, overcoming drug resistance mechanisms and enhancing tumor penetration [16–18]. The important for the treatment of peritoneal surface malignancies that that has limited response to systemic chemotherapy since no current approved Food and Drug Administration (FDA) drugs for intraperitoneal treatment [19]. The following section will provide an overview of nanoparticle technologies used for intraperitoneal cancer drug delivery.

### Advantages of using nanoparticles as drug delivery vehicles in the peritoneum

Nanoparticles delivered intravenously or intraperitoneally selectively localize to tumor through passive and active targeting mechanisms. Passive accumulation of nanoparticles is mediated by enhanced permeability and retention effect (EPR), which relies on imperfections in tumor architecture for intratumoral accumulation, and retention due to diminished lymphatic recovery by the tumor [20]. Active targeting is achieved through the addition of specific moieties to the surface of nanoparticles that can interact with a wide range of molecular targets to enhance nanoparticle localization to tumors. The availability of biodegradable slow-release formulations and offers the prospect of long-acting intraperitoneal chemotherapy. Xu and colleagues developed a biodegradable Paclitaxel-loaded thermosensitive hydrogel system that demonstrated enhanced cytotoxicity to CT26 cells, residence time upward of 96 hrs compared to 24 hrs for Taxol^®^ and excellent biocompatibility [21].

In addition to rapid clearance, many existing chemotherapeutic agents effective for IV treatment of peritoneal malignancies are either highly hydrophobic or toxic to peritoneal tissues [22–25]. Sequestering drugs within nanoparticles allows delivery of highly hydrophobic drugs into the peritoneum and minimizing toxicity to healthy tissues by eliminating solvents or carriers. The use of nanocarriers also increases the intraperitoneal and intratumoral concentration of drugs as well as the maximize the tolerated dose compared to conventional formulations [26]. For example, nanoparticle formulation of paclitaxel (Nanotax^®^) at doses up to 275 mg/m^2^was delivered intraperitoneally with no Grade 2, 3, or 4 neutropenia compared to Grade 4 neutropenia at 175 mg/m^2^ of standard formula of paclitaxel [27]. Differences in sampling methods and data reporting preclude the comparisons of efficacy and pharmacokinetics of standard versus nanoparticle drug formulations. Available pharmacokinetics data from selected studies are summarized in Table 1.

**Table 1:** Comparisons of efficacy and pharmacokinetics of standard versus nanoparticle drug formulations

Drug	Exposure Time	C_max IP_ mg/L	C_max Plasma_ mg/L
Paclitaxel [28]	2 hours	46.6^1^	0.112
Nab-PTX [36]	Not reported	40.62^2^	0.138
Nanotax^®^ [120]	30–60 minutes	5.72^3^	0.004

^1^Dose of 175 mg/m^2^ measured at 2 hours in 12 patients.

^2^Doses of 35–112.5 mg/m^2^ measured at 0, 1, 2, 4, 6, 8, 24 and 48 hours over 1 cycle (Days 1 and 15) in 8 patients.

^3^Doses of 50–275 mg/m^2^ measured at 2 hours in 13 patients.

While most of the literature on the intraperitoneal use of nanoparticles has focused on nanoparticle delivery of chemotherapeutic or imaging agents, some studies have explored the possibility of using nanoparticles as palliative therapy to prevent peritoneal adhesions and scarring [29]. Such of these studies showed that tanshinone IIA loaded nanoparticles delivered intravenous (IV) was successfully prevented peritoneal adhesions in rats by upregulating fibrinolysis [30]. Alternatively, developing of tissue plasminogen activator loaded with thermosensitive hydrogel exhibited an excellent anti-adhesion effect in a rat model that repeated-injury of intraperitoneal adhesions [31].

### Types of nanocarriers

A variety of materials including polymers (dendrimers, nanospheres, nanoparticles, polymer-drug-conjugates, injectable and implantable depots), lipids (liposomes, solid lipid nanoparticles), metals (quantum dots, gold nanoshells), and carbon (buckyballs, nanotubes) are available for nanoparticle synthesis. Selection of material depends on the desired properties of the final nanoparticle as well as characteristics of the intended drug cargo such as hydrophobicity and susceptibility to enzymatic degradation. Polymers and lipids have received special attention for use in drug delivery system because of their excellent biocompatibility and versatility as well as their promising results from *in vivo* studies of intraperitoneal delivery [32, 33]. The list of nanocarriers that have been used so far in medicine is summerized in Table 2.

**Table 2:** Types of nanocarriers used in clinical applications

Type of nanocarriers	Examples	Advantages	Application
Naturally polymers [34]	- heparin	- highly biocompatible	- advanced prostate cancer
	- chitosan	- biodegradable	- non-small cell lung cancer and breast cancer [35]
	- gelatin	- non-toxic	
	- hyaluronate	- non-immunogenic	- intraperitoneal treatment [36]
	- albumin		- peritoneal metastasis in gastric cancer [37]
Synthetic polymers [38]	- PEG	- biodegradable	- imaging and therapy [39]
	- PLGA	- biocompatible	- drug delivery in the peritoneum [41]
		- non toxic	
		- modifies the surface of a variety of nanoparticles	
		- improves *in-vivo* stability	
		- preventing opsonization and phagocytosis	
		- diminishing clearance by the reticuloendothelial system [39, 40]	
Liposomes [43]		- approved by the FDA [42]	- ovarian cancer [48]
		- susceptibility to opsonization and clearance by the reticulo-endothelial system	- drug encapsulation and loading
		- increase likelihood of lodging in the lymph nodes	- controlled rate of drug release [49, 50]
		- propensity to liposomal vesicle destabilization [44, 45]	
		- ready availability	
		- non-toxic,	
		- biodegradable	
		- unique structure that creates two separate compartments for entrapment for both lipophilic and hydrophobic compounds [46, 47]	

PEG - polyethylene glycol; PLGA - poly(lactic-co-glycolic acid).

### Overview of nanoparticle types

The shape and architecture of nanoparticles determine how the drug is affixed to the carrier and transported in the body. Drugs can be absorbed onto, encapsulated within, conjugated to or absorbed into nanocarriers. Drugs can also be loaded into nanoparticles using self-assembling hydrophobic/hydrophilic micelles, direct conjugation via chemical synthesis, self-assembled. Drug loaded nanoparticles have several mechanisms to release of the encapsulated material, including light triggers (near-infrared, ultra-violet), thermal stimulation, magnetic guidance, ultrasound, electrical stimulation and endogenous gradients which can be dependent on pH, enzymatic concentrations or redox potential [51, 52]. Table 3 provides the brief descriptions of nanoparticles with peritoneal applications.

**Table 3:** Types and peritoneal cancer applications of nanoparticles

Type	Description	Applications
Liposomes	Colloidal particles composed of phospholipids. On contact with water, hydrophobic and hydrophilic components	Mirvetuxumab soravansine in combination with pegylated liposomal doxorubicin in adults with folate receptor alpha positive primary peritoneal cancer [53] Intraperitoneal delivery of paclitaxel [54]
Nanospheres	Solid spherical structures composed of a matrix into which a drug is disbursed	IP injection for *in vivo* imaging of intraperitoneal tumors [55]
Micelles	Self-assembling spherical structures with an inner hydrophobic core and an outer hydrophilic shell	IP delivery of paclitaxel-loaded micelles to treat ovarian cancer [56]
		Prevention of peritoneal adhesions [28]
Injectable and implantable depots	Macroscale deposits of liquid or gel-like matrix containing a therapeutic agent or nanoparticles loaded with a therapeutic agent	Intraperitoneal chemotherapy with extended residence time and slow release
		Hyaluronic acid-based hydrogels for delivery of paclitaxel to treat peritoneal tumors [57]
		Hyaluronic acid-based hydrogels for delivery of cisplatin for treatment of disseminated gastric cancer [58]
		Prevention of peritoneal adhesions [59, 60]
Expansile Nanoparticles	Environment-responsive spheres that expand and release contents in response to a programmed stimulus such as pH	pH-triggered intraperitoneal delivery of chemotherapy [61]
		Paclitaxel loading for intraperitoneal delivery with extended release time [61, 62]
Gelatin nanoparticles	Solid spheres composed of natural, non-toxic biopolymer in micro- or nano-size range	Gelatin nanoparticles for paclitaxel delivery in mouse model of disseminated peritoneal cancer [63]
		Suppression of peritoneal fibrosis using siRNA-conjugated gelatin microspheres [64]
Bioadhesive nanoparticles	Polylactic acid-based copolymer nanoparticles	Adhesion to proteins allows longer residence time in the peritoneum [65]
Mesoporous silica nanoparticles	Biocompatible, biodegradable spheres with pores of adjustable sizes allowing drug loading and modifiable drug release rate	Mesoporous silica nanoparticles improve paclitaxel loading and peritoneal residence time [66]

### Challenges in intraperitoneal nanoparticle drug delivery

Nanoparticles face multiple physical and biological barriers on the way to their target. Increasing the number of barriers resulted in diminishing scale of the targets. These barriers include reticular structures, lumens of small capillaries, endothelial covering of vessels, epithelium and stroma of tumors, cell membranes, and nuclear membranes. Additional challenges in the peritoneum include achieving its distribution in the cavity due to the presence of organs, mucus, fluid, effects of gravity, and micro-scale obstacles such as phagocytosis that remove by macrophages and clearance through lymphatics. The distribution of nanoparticles can be physically improved through manipulation of organs and positional changes, adverse effects of mucus and premature clearance. A review of human and animal peritoneal physiology studies reveals several important considerations for the design of intraperitoneal therapeutics including: 1. higher molecular weight and diameter of prolong peritoneal residence time, 2. lymphatic drainage is the major route of removal of intraperitoneally delivered drugs, 3. peritoneal capillary membrane transports water and water-soluble substances through 4 nm pores and proteins through 25 nm pores and 4. negatively charged agents are rapidly cleared from the peritoneum, while positively charged agents exhibit increased retention time [67, 68]. Previously considerable effort has been devoted to develop a nanocarrier that optimized for peritoneal delivery, including manipulation of size, surface charge and PEG coating.

Studies of nanoparticle-mucus interactions reveal that mucus interferes with penetration of nanoparticle through hydrogen bonding, adhesion, and electrostatic interactions [69]. PEG coating has been used to minimize mucus-nanoparticle interaction thereby increasing nanoparticle penetration through mucus [70]. To achieve the target, alternative strategies include conjugation of mucolytic to the nanoparticle surface or the extensive mucin content of mucus has been used. It is important to consider the toxicity effects of intraperitoneal delivery of nanoparticles. These effects include systemic penetration, splenic and liver accumulation, and inflammatory responses in the peritoneum. The safety findings from selected studies of intra-peritoneal (IP) nanoparticle delivery are summarized in Table 4.

**Table 4:** Conclusions from select studies/trials of intraperitoneal nanoparticle safety

Nanoparticle	Study	Year	Results
***Animal studies***			
Microspheres	Kohane at al. [40]	2006	Microspheres induced inflammation Liver and splenic sequestration with foamy macrophages
***Human studies***			
Paclimer microspheres	Phase I Trial [25]	2006	Microspheres induced inflammation; study discontinued
Pegylated liposomal Doxorubicin (with HIPEC at the time of CRC)	Phase I trial [71]	2008	Well tolerated
Nanotax^®^(nanoparticulate paclitaxel	Phase I Trialm [25]	2015	Well tolerated with minimal systemic exposure and reduced toxicity compared to IV paclitaxel

### Surface modifications for targeting of peritoneal malignancies

The search for molecular targets to enhance target the tumor by the nanoparticle has yielded several candidates with potential for clinical utilization. These candidates include folic acid and transferrin which are showing the most effective enhancers [72–76]. An alternative targeting strategy involves is encapsulation of targeted therapeutic agent in non-targeted nanocarrier [77]. Currently, the bevacizumab and pemetrexed only target therapeutics have been used in the peritoneal cavity. Bevacizumab was successfully used as a palliative treatment of malignant ascites in peritoneal carcinomatosis of ovarian origin [78]. Pemetrexed (folic acid targeting drug) used in Phase I trial for treatment of optimally debulked ovarian, peritoneal, and tubal cancers showed lower toxicity and efficacy compare to other chemotherapeutic agents [79, 80].

### Nanoparticles and near infrared (nir) irradiation

Many advances listed for the aforementioned drug delivery systems (DDS) that encourage number of groups to explore the great effect of dual combination of external excitatory source and nanoparticle based DDS [81, 82]. The investigations have entailed to use different types of external energy sources to activate or control the drug release [83]. This approach via the anticancer hyperthermia techniques method has been quickly adopted and focused on the Near Infrared (NIR) Irradiation to take the therapeutic advantages of specific effects to deliver the nanoparticles [84, 85].

NIR irradiation identified 200 years ago and its used in nanomedical since 1990s [86]. NIR irradiation represents specific electromagnetic wave that exhibiting both wave and particle properties to be strongly absorbed by water, hemoglobin and myoglobin. As demonstrated in experimental animal models, this type of irradiation noninvasively delivers energy to cytochrome C oxidase by stimulating the respiratory chain enzyme (Complex IV) and leads to increased adenosine triphosphate (ATP) production [87, 88]. The most important therapeutic useful effect in targeted tumor involves hyperthermia implementation when the nanoparticles used for phototherapy-based therapeutic strategies [89–93]. The main specific effects of NIR irradiation and nanoparticle implantation are favored and/or considered most important to cancer treatment are summarized in Table 5.

**Table 5:** Data from *in vitro* and *in vivo* studies described the main specific effects of Near-infrared (NIR) irradiation use combined of nanoparticle implementation potentially prospectively that useful in HIPEC and PIPAC

Selected specific effects of the NIR irradiation use combined with nanoparticle implementation	Possible practical application	Reference
**Hyperthermia**	The possible usage of NIR induced hyperthermia for example using the phenomenon of surface plasmon resonance effect to destroy only cancer cells.	Chatterjee DK et al. 2011 [91] Margheri G. et al. 2016 [92] Huff TB et al. 2007 [93]
**Fully localized (targeted) therapy**	The use of NIR and selected nanoparticles allows for fully targeted anticancer treatment i.e. in intratumoral nanospheres–cytostatics systems application or selective anatomical distribution with subsequent radiation. Such therapy could prospectively acts more sparing on healthy cells and tissues.	Gupta S. et al. 2012 [94] Hong C. et al. 2011 [95]
**The possibility of multiple therapeutic interventions**	The use of a representative group of nanoparticles allows the multiple NIR intervention in case of the planed interval treatment or rapid progression.	Krishnan S. et al. 2010 [96] Chitgupi U. et al. 2017 [97]
**Radiation dose enhancement**	Some authors suggested that selected nanoparticles such as gold nanoparticles could be used to enhance the radiation dose with good anticancer effects.	Hainfeld JF. et al. 2004 [98] Chen CW. et al. 2014 [99]
**Precise control over the released dosage**	In some applications of nanoparticle based DDS the amount of the released anticancer molecules could be well-tuned by altering the time duration and intensity of NIR light exposure. This specific effect and property is especially important in practical application when the depot form of nano-DDS is used	Bagheri A. et al. 2016 [100] Carling CJ et al. 2015 [101]

There have been only a few published papers that have fully referred to the potential use and initial experimental effects of nanoparticles and NIR for HIPEC and none for PIPAC. One of the first publications addressing this topic is detailed review suggested that the NIR/nanoparticle concept could improve HIPEC [102]. They further discussed the current three main types of nanoparticles including: carbon nanotubes, metal and magnetic nanoparticles. Moreover, Single Walled Carbon Nanotubes (SWNT) is more efficient than gold nanoshells when its stimulated by infrared light and when SWNT stimulated by radiofrequency induces much higher temperature.

Wu *et al.* tested nanoparticle-induced intraperitoneal hyperthermia and targeted photoablation on ovarian cancer using ID8 cells and non-scid mouse with artificially induced PC using ID8-luc cells (C57BL/6 origin) [103]. They used pegylated silica-core gold nanoshells (pSGNs) with external near-infrared (NIR) laser irradiation and found that repeated photothermal treatment can effectively eliminate intraperitoneal tumors. Furthermore, they found that conjugation of pSGNs with anti-human CD47 monoclonal antibody enhanced targeted intraperitonal anti cancer therapy. Their findings raised the possibility of repeated non-invasive photoablative therapeutic interventions after initial HIPEC. The number of promising nanomaterials for NIR-induced hyperthermia increased but their cytotoxicity and systemic impact need more evaluation [104].

### Nanoparticles in perioperative diagnostic

Over the last few years there are improvements in the treatment of patients with peritoneal metastases (PM) as well as diagnosis of the advance stage of the disease by using the fluorophore nanoparticles. The new era of surgical guide with molecular navigation helped in detection of tumor in lymph nodes, vessels and vital structures and lead to improve the surgical precision. The fluorphores are visible in the near-infrared light range (700–900 nm) [105]. It can be visualized using a special camera with light emitting diodes (LED) when it injected into the patient [106]. The LED produce is a beam with near-infrared wavelength to which the fluorophore responds and emits another wavelength of near-infrared light that can be detected with a camera [107]. Many new nanoparticles have been developed that are widely used in sentinel node biopsy, visualization of gastrointestinal structures, ureter visualization, and tumor visualization as well as peritoneal spread [108–112]. Real time visualization of fluorphores may help in optimizing the planning of surgical procedures, the protection of important vital structures such as the ureter or common bile duct, visualization of blood vessels or nerves, obtaining a negative resection margin and detection of small tumor deposits. The limitation of NIRF (near-infrared fluorescent) guided surgery is small detection depth (about 5–8 mm) [107]. Currently, the mostly widely fluorophores that approved for use in humans are indocyanine green (ICG), methylene blue (MB) that represents some NIRF properties, 5-aminolevulinic acid (5-ALA), and fluorescein. In cytoreductive surgery (CRS) and HIPEC, we can use NIRF-guided nanoparticles like MB for visualization of important structures such as the ureter, which is extremely important in reoperations [110]. NIRF nanoparticles are also used in the detection of peritoneal spread. One study has used tumor specific fluorescence imagining to target over expressed folate receptor-α in ovarian cancer and more tumor deposits were visualized using NIRF nanoparticle than with the naked eye [111]. The visualization with targeted NIR fluorphores in CRS and HIPEC procedures may result in more efficient cytoreduction due to more precise resection of even the smallest tumor nodules [111]. Another study from the same team also analyzed the sensitivity and diagnostic accuracy of α(v)β(3)-integrin-targeted NIRF compound in ovarian cancer and found it has 95% sensitivity, 88% specificity, and 96.5% diagnostic accuracy [113]. Optimization of molecular imaging that combines immunological probes with fluorophores in order to improve cancer detection is actively being pursued [114]. Currently, there are clinical 3 trials to evaluate NIRF technology for the detection of peritoneal spread (NCT02032485, NCT01982227, NCT 01834469). The pilot study results from the first trial (NCT02032485) showed evaluation of NIRF imagining in peritoneal metastases detection due to colorectal cancer [115]. Intravenous indocyanine green (ICG) was intraoperatively applied to 63 of 78 peritoneal resected nodules in 14 patients. These data showed that the fluorescence and final pathological examination are 84% malignant and 16% benign. Moreover, in 4 of 14 (29%) patients surgery the fluorescent detection was adjusted because of the additional PM not detected by naked eye visualization and palpation. The data from the pilot study concluded that ICG fluorescence imaging appears to be particularly useful for non-mucinous PM of colorectal origin. The visualization of PM in gastric cancer mouse model using ICG combined with antibodies against CEA or EGFR was reported [112]. The potential of near infrared photoimmunotherapy in peritoneal carinomatosis was reported in ovarian origin of mouse model [116]. The utility of ICG with other probes was demonstrated using a liposomal synthesized ICG liposomal derivative [117].

Another potential 5-ALA fluorophore demonstrated in PM evaluated the best pre-operation application time and the dose for intraoperative detection of peritoneal metastases in ovarian cancer patients [118]. In addition, 5-ALA was used for detection of staging of laparoscopic gastric cancer surgery [119, 120]. These studies evaluated the peritoneal visualization after chemotherapy by conventional laparoscopy in 12 of 38 patients with advanced gastric cancer and peritoneal metastases. Additional 4 patients were positive for peritoneal metastases with laparoscopy and 5-ALA visualization and interestingly 3 of these 4 patients were negative by peritoneal washing cytology analysis [120]. Combination of 5-ALA fluorescence detection with molecular analysis of specific genes that mediate 5-ALA transport, such as peptide transporter PEPT1 (ALA influx transporter). PEPT1 was overexpressed in tumors suggest that evaluation of the expression of PEPT1 may help in the selection of patients who will benefit from 5-ALA visualization of tumor deposits [121].

### Nanoparticles as anticancer dressing’s, hydrogels and as active beads used for optimal recurrence prevention

The preclinical studies from using of novel DDS systems based on nanoparticles for the treatment and diagnosis in the peritoneal surface malignancy could lead to new applications for NP's such as direct chemotherapeutics delivery into the compartment created after resection or before or after HIPEC and PIPAC procedure [122]. Fan *et al.* demonstrated antitumor effects of docetaxell/LL37 in tumor bearing mice and loaded thermosensitive hydrogel nanoparticles in PC of colorectal origin [123]. In addition, polymer-lipid biodegradable depot (PoLigel) used in mouse model of ovarian cancer for sustained intraperitoneal chemotherapy resulted in reduction of tumor burden supporting the notion that nanomaterials loaded with cytostatic could be used to enhance anti-tumor effects and prevent or mitigate recurrence [124].

Various advances in DDS for intraperitoneal therapy such as implants and injectable depots to extend the residence time of chemotherapeutic agents in the peritoneal cavity have been discussed [125]. Based on nanofibers, carbonaceous nanomaterials and polycaprolactone materials were evaluated and termed nano- desings or hybrid beads with anticancer properties [32, 122, 126]. The honokiol nanoparticles and called the forms thermosensitive hydrogel composites have been also used [127]. The terminology for DDS will be depend on number of diverse factors including: types of nanomaterial (i.e., biodegradable-active carriers of drug), pharmacodynamics and pharmacokinetic knowledge of the material; the particular programmable smart targeted drug delivery device [128, 129] and the intraoperative application(s) of particular depot.

Currently there are two primary direction to use the array of available nanoparticles to extend the residence time of cytostatic in the peritoneal cavity and improve their targeted influence [122, 130]. First is to be used it before HIPEC to support CRS and prospectively for protection with biopsy for histopathology and resections performed laproscopically before PIPAC procedure. Second is to be used it after completion of HIPEC or PIPAC during the last perfusion/spraying cycle or after the washing procedure [32]. The biodegradable depot solutions available are summarized in Table 6. The tumors following resection after organ sparing of non-anatomical resections parenchymal organs are detected on the liver surface [32]. The material used in these procedures should be sufficiently durable to withstand all subsequent HIPEC or PIPAC procedures. The second approach is not required any special development of materials after HIPEC or PIPAC to avoids exposure to circulating/sprayed liquid or heat [32]. The large challenge is to find the depot forms that meet specific product characteristics and important following requirements; full cytotoxicity without side effects, cytostatic distribution, planned biodegradation and physicochemical stability. Nonetheless, it is widely recognized that drug delivery by nanoparticles is highly promising and will likely follow the rigorous of preclinical and clinical studies develop in HIPEC and PIPAC [32, 134–136].

**Table 6:** Potentially suitable materials possible to use in the future construction of depot supporting tools for HIPEC and PIPAC

Type of used material	Type of experimental intervention	Reference
Pegylated silica-core gold nanoshells (pSGNs) *in vivo* with external near-infrared (NIR) laser irradiation.	Experimental nanoparticle-induced intraperitoneal hyperthermia and targeted photoablation in treating ovarian cancer.	Wu et. al. [103]
Thermosensitive hydrogel system (PTX/PECT(gel)) assembled by PTX (paclitaxel)-loaded amphiphilic copolymer	Thermosensitive hydrogel system used in experimental for sustained intraperitoneal chemotherapy of peritoneal carcinomatosis.	Xu et. al. [21]
Nanovehicles based on anti-CD133 antibodies bioconiugated to carbon nanotubes loaded with platinum (Pt) –prodrugs	Nanovehicles used as a novel target strategy for hyperthermic intraperitoneal chemotherapy on mouse melanoma B16 PC model.	Nowacki et. al. [32]
Curcumin loaded polymeric micelles (Cur-M)	Anti-Tumor Activity of Curcumin by Polymeric Micelles in Thermosensitive Hydrogel System tested in Colorectal Peritoneal Carcinomatosis Model	Zhang et al. [131]
Paclitaxel-loaded pH-responsive expansile nanoparticles (Pax-eNP)	Expansile nanoparticles used in *in vitro* and *in vivo* murine models of malignant peritoneal mesothelioma	Colson et al. [142]
Paclitaxel loading nanoparticle (PLA) by ultrasonic emulsification	Nanoparticles tested *in vitro* on rat ovarian carcinoma cells and *in vivo* on PC induced in F344 rats	Lu et al. [132]
Combination of 5-fluorouracil (5-FU) loaded polymeric micelles and cisplatin (DDP) in biodegradable thermosensitive chitosan (CS) hydrogel	Nanosystems tested on colorectal peritoneal carcinomatosis mouse model	Yun et al. [133]

### *In vitro, in vivo* and clinical studies

Nanoparticles have a potential important role in cancer therapy. However, utilization of nanomaterials in HIPEC is a new concept. Most of the research with the application of nanoparticles in peritoneal carcinomatosis treatment has been without the use of hyperthermic conditions. The registered clinical studies using nanoprticles in peritoneal carcinoma are summarized in Table 7. Using nanoparticles in ovarian cancer and associated peritoneal carcinomatosis is the primary interest. Hijaz *et al.* evaluated the preclinical use of cerium oxide nanoparticles conjugated with folic acid (NCe-FA) for treatment of ovarian cancer. Folate receptor-α has been reported to be overexpressed in ovarian cancer. Exposure of ovarian cancer cells to NCe-FA led to the intracellular accumulation of drug and inhibited cell proliferation. Mice treated with NCe-FA compared with NCe exhibited decreased in tumor burden without toxicity. In addition, combined treatment of mice with cisplatin enhanced the anti-tumor action of NCe-FA [137].

**Table 7:** Registered clinical studies using nanoprticles in peritoneal carcinoma

Conditions	Intervention	Enrollment	Phase	Number
Fallopian tube carcinoma Primaryperitonealcarcinoma Recurrent ovarian carcinoma	Nab-PTX	51	II	NCT00499252
Peritoneal carcinoma	Nanotax	22	I	NCT00666991
Ovarian cancer Peritoneal cavity cancer	Nab-PTX	27	I	NCT00825201

The cytotoxicity of another folate receptor-targeted formulation with paclitaxel [folic acid-coupled PEGylated nano-paclitaxel liposome (FA-NP)] was evaluated in paclitaxel-resistant SKOV3/TAX ovarian cancer cells and in murine model of peritoneal ovarian cancer. FA-NP overcame paclitaxel-resistance in ovarian cancer raising the possibility of the FR-targeted chemoagents might prolong the survival in patients with drug-resistant ovarian cancer [138]. Another group with similar findings underscore the potential of folate-targeted nanoparticles containing chemoradiotherapy for treating ovarian peritoneal metastasis [139]. Most ovarian tumors and peritoneal implants express the CD44 receptor, a mediator of drug resistance that is associated with unfavorable prognosis. Hyaluronic acid (HA) is the ligand for CD44 promotes the proliferation, migration and invasion of cancer cells. Based on this observation HA has been evaluated for CD44-targeted chemotherapy in ovarian cancer. Injection of HA-based Paclitaxel (PTX)-loaded nanoparticles significantly reduced tumor burden compared to conventional PTX, thereby underscoring the promise of HA-based nano-system for delivering PTX [140].

Wang *et al*. evaluated the preclinical effect of selenium (Se) nanoparticles in mouse model of peritoneal carcinomatosis. The Se nanoparticles were administrated intraperitonally prior to the injection of high malignant H22 hepatocarcinoma cells into the abdominal cavity. Overall, their findings demonstrated that Se nanoparticles induced of reactive oxidant species (ROS), exhibited potent anti-tumoral effects without induction of host cytotoxicity indicate the possibility of high risk to patients with PC [141].

A number of studies have investigated the application of paclitaxel (PTX) nanoparticles for peritoneal cancer treatment. Polymeric pH-sensitive nanoparticles engineered to slowly release PTX (Pax-eNP) at endosomal pH (pH ≤ 5), thereby allowing for extended drug action to deliver the chemotherapeutic drug in mouse models of malignant peritoneal mesothelioma. The DDS exhibited prolonged intraperitoneal residence time, enhanced cellular uptake and increased tumor affinity. It was approved to be effective against a mesothelioma cell line (MSTO-211H) *in vitro*, and simultaneous treatment with DDS of the tumor potently decreased tumor burden, initial intraperitoneal tumor implants and extended survival [142]. Pax-eNP was also evaluated in rat xenograft model of pancreatic peritoneal carcinomatosis using Panc-1-cancer stem cells. They found that cancer stem cells are responsible for disease recurrence, therapy resistance and metastatic phenotype. Although nanoparticles loaded with PTX inhibited tumor growth to the same extent as PTX alone but it showed fewer side effects [143]. The ability of liposome-encapsulated paclitaxel (Nano-Taxol) to influence the stemness phenotype and metabolic reprogramming of a paclitaxel-resistant cell line was investigated. Intraperitoneal delivery of Nano-Taxol in mouse xenograft model controls of tumor growth compared to standard treatment with Taxol® (intravenous delivery) [144].

Promising preclinical studies have led to introduction of nanoparticles in clinical practice. A number of reported advantages of using albumin nanoparticles loaded with PTX (nab-PTX) include: ability to delivery of higher dose of paclitaxel; obtain higher intratumor concentration of paclitaxel; easy administration and avoidance of Cremophor EL medium and related toxicity [145]. Nab-paclitaxel was used to treat 47 patients with platinum- and taxane-resistant ovarian cancer. Data showed the persistent or progressive disease following primary chemotherapy or recurrence within six months of completing treatment. Nab-PTX demonstrated significant clinical efficacy and was well tolerated [145]. Nab-PTX was also evaluated in patients with recurrent epithelial cancer of the ovary, fallopian tube, or peritoneum. Among the 44 patients who underwent treatment, 15 demonstrated complete and 13 partial response [146]. Nanotax^®^, a sterile nanoparticulate paclitaxel powder was used in 21 patients with peritoneal malignances and its intraperitoneal administration was well tolerated by patients with slide side effect. Use of DDS showed significant and prolonged concentrations of paclitaxel in peritoneal fluid [147]. Although there are small numbers of registered clinical trials evaluating nab-PTX for peritoneal carcinoma treatment, this area of clinical investigation is nonetheless highly promising (Table 7). Currently, clinical studies are evaluating the use of intraperitoneal administration of nanoparticles alone, without induction of hyperthermic conditions. Combining nanotherapy with hyperthermia could potentially enhance peritoneal carcinoma treatment and important step in the evolution of the treatment of peritoneal carcinomatosis.

## SUMMARY AND CONCLUSIONS

The number of HIPEC and PIPAC administrations performed yearly is growing systematically worldwide. Simultaneously, the number of fully qualified and reference centres offering these therapies is fortunately increasing as well [148, 149]. There are also many new studies related to development of these techniques being conducted such as those aimed to improve the surgical and technical aspects of the procedures to optimize the patient selection [150–152]. The large investment of resources correlates naturally with the emmerging social need and register the number of patients suffering from peritoneal carcinomatosis [153]. Currently, the HIPEC is fully recommended and recognized for treatment to increase patient survival with improvement this kind of therapy using nanomedicine [154, 155].

In the current review, we summarized the current use, developing concepts and strategies of nanoparticles *in vitro*, *in vivo* and in clinical studies and their potential impact on HIPEC and PIPAC. The use of nanoparticles as novel drug delivery systems and the evaluation of diverse materials in various applications has been area of concerted investigation pushing the frontier of cancer diagnostics and therapeutics. Developments of nanoparticle-based DDS have occurred with carbonaceous origin nanoparticles and gold nanoshels. Many novel advances have been made using of nanoparticles together with NIR irradiation for enhance drug delivery and improvements of perioperative diagnostics. The implementation of special beads and hydrogels in the construction of nanovechicles loaded with specific drug combinations and conjugated with select antibodies for targeted anti-cancer treatment has generated significant excitement.

In conclusion, there is large a number of innovative and translational studies to advance using of nanotechnologies as permanent treatment for peritoneal carcinomatosis. Several preclinical and early clinical studies using nanoparticle in intraperitoneal chemotherapeutic methods in HIPEC and PIPAC provided a new evidence of its effectiveness for treatment patients with PC. Most of the research projects have been carried out *in vitro* and *in vivo* models has been invaluable prior to clinical translation. The key issues of non-tumor cytotoxicity and product safety need to be fully addressed prior to nanoparticle implementation. We hope the new direction of therapy using advance nanotechnologies as nanoparticle-based chemotherapy will be more effective for treatment patients with peritoneal carcinomatosis.

E. McCabe, D. Cortes Guiral, K. Polom, G. Dakwar, K. Pietkun, B. Zegarska, M. Pokrywczynska, T. Drewa, F. Roviello and W. Zegarski: Data analysis and interpretation, drafting the article.
